# Surgical treatment results in patients with defects of the integumentary tissues of the trunk and limbs of mechanical origin

**DOI:** 10.25122/jml-2022-0019

**Published:** 2022-11

**Authors:** Olena Vasylivna Ponomarenko, Ludmila Nylsivna Serhieieva, Kyrylo Yuriiovych Parkhomenko

**Affiliations:** 1 Department of Medicine of Catastrophes, Military Medicine and Neurosurgery, Zaporizhzhia State Medical University, Zaporizhzhia, Ukraine; 2 Department of Medical Physics, Biophysics and Higher Mathematics, Zaporizhzhia State Medical University, Zaporizhzhia, Ukraine; 3 Department of General Practice Family Medicine and Internal Medicine, Kharkiv National Medical University, Kharkiv, Ukraine

**Keywords:** treatment effectiveness, skin, wound surface, integumentary tissue defects, reconstructive interventions

## Abstract

This study aimed to develop and implement a universal method for the quantitative assessment of treatment effectiveness in patients with skin and underlying soft tissue defects of the trunk and extremities. The study involved 242 patients, including 46 patients with upper extremity injuries, 179 with lesions of lower extremity tissues, and 17 patients with defects of the integumentary tissues of the trunk. The greatest treatment effectiveness was observed in patients with upper limb injury: excellent result – 60.0%, good – 33.3%, unsatisfactory – 6.7% of patients. In the group of patients with lower extremity injuries, an excellent result was recorded in 19.6% of cases, good (58.1%), satisfactory (15.1%), and unsatisfactory in 7.2% of patients. In patients with trunk injuries, an excellent treatment result was obtained in 23.5%, good – 35.5%, satisfactory – 23.5%, and unsatisfactory – 17.6%. The universal quantitative method for evaluating treatment effectiveness in patients with various types of damage to the trunk and extremities tissues was proposed. This method makes it possible to objectively determine the level of medical service provided to each patient, which is of great importance in the context of medical service reorganization in the state.

## INTRODUCTION

Modern injuries are characterized by high-energy action of the traumatic factor and simultaneous damage to several anatomical structures [1–5]. Of these, more than 48.9% are injuries associated with blood vessels, nerves, osteoarticular apparatus, and tissue defects, which lead to long-term or persistent disability of the injured in 30% of cases [6–9].

However, despite the constantly high level of injuries and their significant variety, there are sporadic data on the algorithm for integumentary tissue recovery and assessment of treatment effectiveness in the literature [10–14]. However, the existing assessments are qualitative, which makes it impossible to compare the results of provided medical care in practical medicine [15–17].

The wide variety of techniques using plastic material to close wound defects allows the surgeon to choose a technique that sometimes might not be appropriate. [18–22]. In addition, the lack of a standardized approach to restoring integumentary tissues depending on the depth and extent of damage to different anatomical structures of body segments of traumatic origin reduces the final efficiency of medical care [23–26].

At the same time, inadequate assessment of damaged tissues, as well as an untimely and inadequate attempt to restore them, should be recognized as one of the reasons for unsatisfactory treatment results, which determines the relevance of the topic studied [27–29].

The above issues are important for assessing the results of medical practitioners' activity, especially in the context of the reorganization of the health care delivery system in Ukraine (Law of Ukraine “On State Financial Guarantees for Public Health Services” No. 2168-VIII of 10/19/2017).

This study aimed to develop and implement a universal method for the quantitative assessment of the effectiveness of treatment results in patients with skin and underlying soft tissue defects of the trunk and extremities.

## MATERIAL AND METHODS

The study involved the examination and treatment of 242 patients with defects of integumentary tissues (DIT) of the trunk and limbs of mechanical origin, which were divided into 3 groups according to the plan.

The first group included patients with lower limb tissues injuries – 179 (74.0%), the second – upper limb – 46 (19.0%), and the third group – injuries of the integumentary tissues in the trunk region – 17 (7.0%).

The main factor for including patients in the study was the lack of opportunities for primary wound healing or DIT formation due to secondary healing, namely – a large area of damage, the presence of non-viable tissues in the wound, infection, and consequently, the development of surgical infection and wound surface (WS) formation, which could not be restored by standard surgical methods.

Criteria for inclusion in the study were male or female patients over 18, diagnosed with a defect of the skin and underlying soft tissues of the trunk and extremities, who needed to restore the shape and function of the body. The study did not include patients according to the following criteria: age less than 18 years, defects in the region of face and head, ulcerative defects caused by chronic vascular or neurological disease, and which were the result of purulent-inflammatory diseases or malignant skin tumors.

The distribution of patients in each group was carried out depending on the area, depth and extent of tissue damage into four types of lesions. Type I lesion (n=44 in three groups) – patients with limited (up to 1% of body surface area) area of damage to the skin and underlying tissues to the deep fascia, type II lesion (n=67 in three groups) – patients with large (more than 1% of surface area) WS and damage of soft tissues below the deep fascia, type III lesion (n=90 in three groups) consisted of patients who received DIT together or due to damage to the osteoarticular apparatus system, type IV lesion (n=41 in three groups) – patients with combined or multiple trauma accompanied by damage to bones, tendons, muscles, great vessels, nerves, partial or complete separation of the limb.

Most patients treated were of working age – 137 (56.6%). There were many patients in the groups of late adulthood and old age – 75 (31%), which was due to home injuries. The majority consisted of men – 156 (64.5%). There were 86 (35.5%) women in the study. Characteristics of patients and variants of tissue damage are shown in Table 1.

**Table 1:** Characteristics of patients in study groups.

Indicator	Upper limb (n=46)	Lower limb (n=179)	Trunk (n=17)	Total (n=242)
**Gender, (n/%)**				
Male	43/93.5	97/54.2	16/94.1	156/64.5
Female	3/6.5	82/45.8	1/5.9	86/35.5
**Age, years**	54.5	46.8	38.8	45.7
**Type I lesion (n/%)**	13/28.4	31/17.3	-	44/18.2
**Type II lesion (n/%)**	7/15.2	56/31.3	4/23.5	67/27.7
**Type III lesion (n/%)**	12/26.1	75/41.9	3/17.7	90/37.2
**Type IV lesion (n/%)**	14/30.5	17/9.5	10/58.8	41/16.9
**The number of postoperative complications (n/%)**	4/8.7	13/7.3	3/17.6	20/8.3

The study groups were homogeneous in terms of the localization criterion and comparable in damage type structure. All patients underwent physical examination, including uninjured limbs and trunk, as a possible donor site. The size of the wound defect was determined in cm^2^. The wound surface of up to 1% of the patient's body surface area was estimated as limited, and over 1% – was estimated as large.

For the examination of patients, along with the examination of related specialists, standardized general clinical laboratory methods (general blood test and urinalysis, biochemical blood test, coagulogram, cultures of wound contents to identify pathogenic microflora, sensitivity to antibacterial drugs and to control the microflora of the wound in dynamics) and instrumental research methods (ultrasound scan of the organs of the thoracic and abdominal cavity, lesser pelvis, retroperitoneal space, soft tissues, chest radiography, electrocardiography, multispiral computed tomography of thoracic and/or abdominal cavity if necessary, endoscopic examination of the digestive tract and respiratory system organs, echocardioscopy if indicated) were used.

The comprehensive program for examining the damaged area consisted of studying regional macrohemodynamics using ultrasound duplex scanning (USDS).

Microhemodynamics assessment was carried out according to the indicators of basal blood circulation in the area of limb injury (perfusion coefficients (K) and degree of its impairment) and on the symmetrical area of a healthy limb.

For an individual assessment of the microhemodynamics of the covering tissues in the area of damage, the integral perfusion coefficient (K) was determined as the sum of the coefficients of the average blood flow, the average fluctuation of perfusion, the relative coefficient of variation, the coefficient of the neurogenic tone of microvessels and the coefficient of the myogenic tone of microvessels.

Analysis of the reparative capabilities of the skin in the injured area was studied using a complex morphological program, which consisted of several stages.

At the first stage of the program, the reparative capabilities of the skin were studied, and keratinocytes were selected and labeled using an enzyme-linked immunosorbent assay kit to quantify CD44. During the operation, a skin sample (up to 1×1 cm^2^) was taken to restore the covering tissues. In the final part of the study, flow cytometry was used to detect active keratinocytes in the damage zone and the degree of their activity (Z). Active receptors of keratinocytes to collagen types I and III labeled with CD 44 were detected, and the Z index was calculated based on the constructed logistic regression model.

In the second stage, the study of collagen formation (determination of the coefficients (Kd) of type I, III collagen deposition) was performed on biopsy-surgical material of skin fragments from the peri-wound zone of the defect. Kd was determined – the coefficient of collagen deposition in the skin as the ratio of the median area of type I collagen deposition to the median area of type III collagen deposition. Immunohistochemical determination of collagen type I and III accumulation was performed using monoclonal antibodies Rb a-Hu Collagen I, Clone RAH C11 and Rb a-Hu Collagen III, Clone RAH C33.

Depending on the type of injury, the patients underwent surgery, which was divided into two stages. The first stage included primary surgical interventions on the injured area – primary surgical debridement (PSD), dissection and drainage of hematomas, and fasciotomy. The second stage was surgery to prepare the WS for the restoration and direct reconstruction of the DIT.

For all types of damage, the share of repair and reconstruction operations (second stage) was: type I lesion – 83.6%, type II lesion – 79.2%, type III lesion – 83.6%, type IV lesion – 74.0% of surgical interventions.

Criteria for evaluating immediate and remote results were engraftment of skin grafts and flaps, removal of DIT, restoration of regional hemodynamics and innervation in the area of injury, elimination of trophic disorders and desmogenic contractures, and restoration of movement stereotype.

The results of treatment were assessed at the time of discharge using functional scales: DASH upper limb impossibility questionnaire [developed by the Institute for Work & Health in collaboration with the American Academy of Orthopedic Surgeons (AAOS) with the support of the American Association of Plastic and Reconstructive Surgeons (2006)] [30]. The condition of the injured lower extremity was assessed according to the LEFS functional scale (Lower Extremity Functional Scale, 1999) [31]. The results of restoring the functional activity of patients with trunk defects were evaluated according to the Functional Independence Measure (FIM).

Statistical processing of quantitative research results was performed on a personal computer using the analysis package in Microsoft Office Excel 2013 and STATISTICA^®^ for Windows 6.0 (StatSoft Inc., license No. AXXR712D833214FAN5). The obtained data were checked for normality of distribution based on the Shapiro–Wilk criterion. In the cases of normal distribution, the arithmetic mean (M) and the standard error of the mean (m) were calculated. The parametric Student's t-test (t) was used to assess the significance of differences between the mean values. p<0.05 was taken for a significant minimum probability of differences.

## RESULTS

### Treatment results in patients with defects of integumentary tissues of the upper extremity

Quality of life was assessed in the postoperative period among 45 patients (out of 46 patients) with posttraumatic defects of the upper extremity. Patient H., born in 1988, case history Nº. 2182 with a diagnosis of traumatic amputation of the left upper extremity; a large granulating wound of the amputation stump of the left upper extremity (type IV lesion) was not studied to assess functional activity.

Thus, the largest number of results, 27 (60.0%), were excellent – primary engraftment of a skin graft or flap and removal of DIT, restoration of 90% of precise and strong types of hand grips, absence of deforming scars and contractures, free functioning of distally located joints (elbow, wrist).

A good result was obtained in 15 (33.3%) patients, and it was characterized by the absence of postoperative complications in the reconstruction area and the restoration of precise and strong hand grips of more than 60%. In some cases (closure of the WS by autodermoplasty), there was a residual deformation that did not affect the functionality of the upper extremity as a whole. Satisfactory results of upper extremity DIT treatment were not noted.

3 patients with type IV lesions had an unsatisfactory result – partial necrosis of the flap with repeated surgery, restoration of precise and strong types of hand grips less than 40% of a healthy hand; there was a partial deformation in the projection of large distal joints (wrist), repeated surgical correction was required. However, an aesthetic effect was achieved.

The use of a differentiated approach to repair damaged integumentary tissues had excellent results in 60.0% of patients, good results in 33.3%, and unsatisfactory results in 6.7% of patients with DIT of the upper extremity.

The functional scale of DASH allowed estimating not only disability but also the efficiency of diagnostic and medical tactics. The effectiveness of treatment outcomes in patients with trauma of the upper extremity is shown in Table 2.

**Table 2:** Evaluation of treatment effectiveness in patients with DIT of the upper extremity, M±m.

Mean values,%			
Type I lesion (n=13)	Type II lesion (n=7)	Type III lesion (n=12)	Type IV lesion (n=13)
92.4±2.0	81.3±5.6	90.3±2.4	72.9±8.2

There was a statistically significant decrease in the effectiveness of treatment for type IV lesions compared to type I (p<0.05) due to severe polystructural tissue damage in this group of injured. In contrast, the effectiveness of type I, II and III lesions did not show a statistically significant difference (p>0.05).

The average treatment effectiveness indicators in patients, taking into account the type of damage to integumentary tissues, shown in the table, indicate the feasibility of the proposed diagnostic and therapeutic tactics not only for type I lesion (due to minimal damage to anatomical structures) but also other more severe upper extremity tissue lesions.

### Treatment results of patients with defects of integumentary tissues of the lower extremity

The condition of the injured lower extremity was assessed according to the LEFS functional scale, which reflected the qualitative assessment in the form of a Likert scale, daily activity.

We proposed a formula for calculating the symptoms of the LEFS scale for quantitative evaluation of results:


$$LEFS=100-\frac{\left[sum\text{ of n answers}\right]}{n}\times 25,$$

where n – number of answers.

After using the formula, the resulting figure corresponded to the percentage of disability. We proposed a 4-point scale, which identified 4 options for the results of rehabilitation of patients with damage to integumentary tissues and other anatomical structures of the lower extremity – excellent, good, satisfactory, and unsatisfactory to qualitatively assess the degree of disability of the lower extremity for practical use.

The data on treatment effectiveness showed excellent rehabilitation results in 19.6% (35/179) of cases among patients with lower extremity injury of type I, II, and III lesions and was characterized by primary skin engraftment or flap and removal of DIT, restoration of regional hemodynamics and innervation in the area of injury, elimination of trophic disorders and, as a consequence, restoration of structural and functional stereotype.

In 2 patients with type II lesions (with consequences of trauma), excellent results were indicated by the absence of desmogenic and arthrogenic contractures, complete absence of pathological scars in the area of injury, and complete restoration of anatomical, functional and aesthetic aspects.

In patients with type IV lesions, there was no excellent result due to the severity of injuries.

The largest number of results on the restoration of the shape and function of the lower extremity – 58.1% (104/179), in the range of good result.

Among the patients with large WS (type II lesion), the number of good results was the largest – 69.6%. This was manifested by the absence of postoperative complications in the reconstruction area with the preservation of the deficit of limb function recovery due to the nature of the traumatic injuries.

The treatment results obtained in patients with type III and IV lesions, with the largest number of structural changes in tissues – 57.4% and 58.8%, respectively, attracted attention.

Examination of patients after 12–36 months noted the absence of desmogenic and arthrogenic contractures, storage of scars in the area of injury, which did not affect the activity of movements, a slight lack of restoration of anatomical, functional and aesthetic aspects.

Satisfactory results were obtained in 15.1% (27/179) of patients – ischemic disorders in the area of postoperative intervention without the need for repeated surgery, preservation of the limb as an organ, or amputation of its distal segments with partial restoration of functional stereotype.

In the group with type I lesion – 22.6%; in group II – 8.9%; in III – 17.3% and group IV – 11.8%.

During the examination of patients after 1 year or more, the absence of arthrogenic contractures, the presence of desmogenic contractures and pathological scars that required surgical correction, partial restoration of anatomical, functional and aesthetic aspects were noted.

Unsatisfactory result – lysis of the graft or necrosis of the complex flap, amputation of the limb due to decompensation of hemodynamics or complete tissue rupture, inability to restore the primary anatomy of the lesion and stereotype of movement, was obtained in 7.2% (13/179) of cases.

Amputation of the limb at the thigh level was performed in 4 patients with type IV tissue lesion.

For the period of observation, the additional signs of unsatisfactory results (desmogenic and arthrogenic contractures, pathological scars in the area of injury) were not detected in 9 patients with complications in the early postoperative period.

The average efficiency values of restorative treatment indicated the maximum number of good results obtained in patients with type I, II, and III lesion of the integumentary tissues of the lower extremity due to the mechanical etiopathogenetic factor – 75.0±3.6%, 76.6±1.9%, and 68.6±2.3%, respectively (p>0.05).

The result of 58.9±9.4% in patients with type IV lesions (p<0.05 compared with types I, II, III) was due to the greatest number of unsatisfactory results of reconstructive interventions associated with severe polystructural damage to the cover, underlying soft tissues, osteoarticular apparatus, great vessels and peripheral nerves of the lower extremity (Table 3).

**Table 3:** Evaluation of the treatment effectiveness in patients with DIT of the lower extremity, M±m.

Mean values,%			
Type I lesion (n=31)	Type II lesion (n=56)	Type III lesion (n=75)	Type IV lesion (n=18)
75.0±3.6	76.6±1.9	68.6±2.3	58.9±6.2

The quantitative assessment of the parameters of household activity and restored disability on functional scales allowed us to determine the effectiveness of the implemented diagnostic and treatment tactics in all clinical groups affected with DIT extremities, as evidenced by the obtained mean values of treatment efficiency.

### Treatment results of patients with defects of the integumentary tissues of the trunk

The results of the restoration of functional activity of patients with DIT of the trunk were evaluated using the functional independence measure by counting the number of points. The patient's integrated index of functional independence (FI) was calculated on a diagnostic scale. The latter reflected the patient's physical and intellectual activity after receiving rehabilitation treatment.

An excellent result (23.5%) indicated the absence of postoperative complications and complete recovery of motor function and social activity. Good (35.3%) and satisfactory (23.5%) results indicated a lack of activity in the postoperative period, primarily due to the severity of the spinal injury and damage to the spinal cord. Unsatisfactory results (partial ischemic necrosis of the complex flap) were obtained in 3 (17.6%) patients – in 1 patient with type III injury FI=30.2%, in 2 patients with injury type IV – FI=29.4% and 27.8%, respectively.

All complications resulted from compression-ulcer trophic defects and required repeated reconstructive surgery.

The mean values of treatment efficacy of trunk DIT were the highest in patients with type II lesions, compared with the results of FI in patients with type III and IV lesions, which was associated with the severity of damage to integumentary tissues, but this difference was not statistically significant due to the small number of patients (p>0.05) (Table 4).

**Table 4:** Evaluation of the treatment effectiveness in patients with DIT of the trunk, M±m.

Mean values,%			
Type I lesion	Type II lesion (n=4)	Type III lesion (n=3)	Type IV lesion (n=10)
-	113.5±13.6	74.7±70.3	71.5±17.8

The mean values of rehabilitative treatment of trunk DIT showed sufficiently high efficiency of the proposed diagnostic and treatment tactics for the fastest recovery of compression and ulcer wound defect, which was the main goal in the group of patients with severe spinal and spinal cord injury for the fastest transition to rehabilitation and improving quality of life in this category of patients.

**Figure 1:**
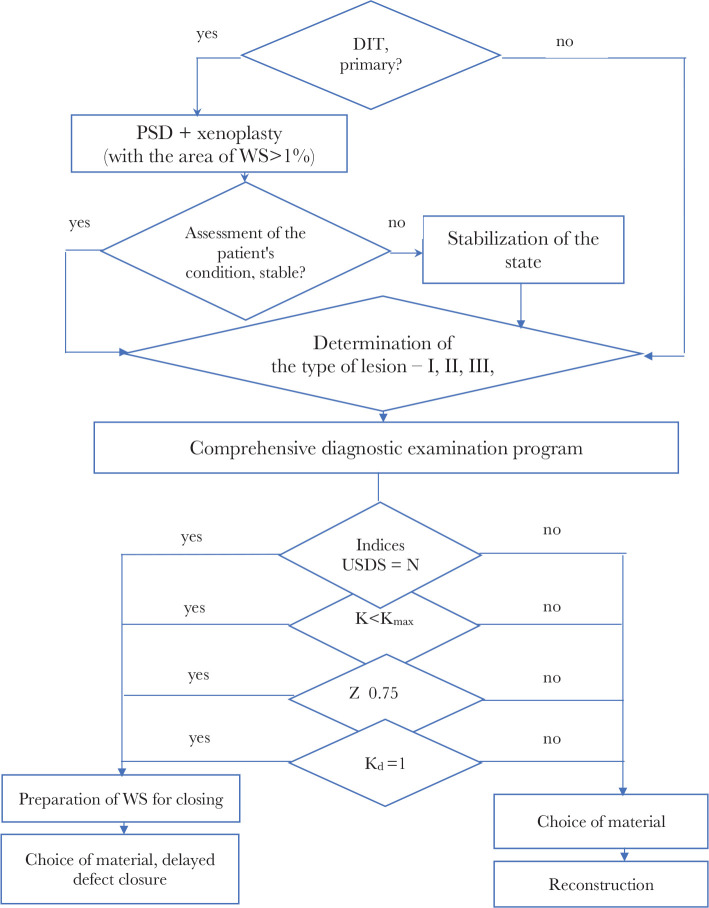
Diagnostic and surgical tactics algorithm for repairing damaged integumentary tissues of the limb after injury. USDS – ultrasonic duplex scanning; K – integral tissue perfusion coefficient; Z – the degree of keratinocytes activity in the damaged area; K_d_ – type I, III collagen deposition coefficient.

The algorithm of diagnostic and surgical tactics for restoring the damaged tissues of an extremity after an injury is developed (Figure 1) based on the data of the complex diagnostic program of research (coefficients of indicators and prognostic scales of ranges of values) and the results of the surgical treatment.

## DISCUSSION

The assessment of the literature and our clinical material convinced us that the earliest possible use of reconstructive surgical technologies from the moment of traumatic injuries is an effective method of treating patients with isolated and multiple injuries of various anatomical structures to restore the shape and function of the body (96.9% of positive result). The use of microsurgical techniques and complex flaps in the acute period of trauma is not always possible and necessary, especially when it comes to severe combined polystructural changes with damage to the great vessels, nerves, bones, and super-large defects of the skin and underlying soft tissues. During such a period, the main task of the surgeon is to close the wound surface quickly to avoid the spread of wound infection and to qualitatively restore the vital structures of the body [32].

The literature describes single studies of the reparative capabilities of the skin, namely the activity of keratinocytes, which are not performed in the context of emergency surgical care [33–35]. The complex diagnostic program developed for studying the reparative capabilities of the skin makes it possible to predict and select the volume of restorative intervention in the early stages after injury. With sufficient individual capabilities of the body to repair integumentary tissues, which we defined as universal quantitative indicators, and the presence of a defect not in functionally active areas (joints), it is not always necessary to expand the volume of the operation, and the main task is the early rehabilitation of the patient and reducing the consequences of body structures traumatization.

This is confirmed by the data analysis of the quantitative assessment of patient's quality of life included in the study groups. Due to this approach, positive results of restorative treatment were obtained within 3 years of observation in 96.0% of patients with type I lesion, 96.78% with type II, 97.87% with type III and 83.76% with type IV lesion of the integument.

## CONCLUSIONS

The complex algorithm of reconstruction of the damaged integumentary tissues depending on the lesion, type, and anatomical, hemodynamic and morphofunctional features of the injury site is proposed, which makes it possible to decide on the use of the reconstruction method in each case. Due to this approach, a positive reconstruction result was obtained in 96.9% of cases. Furthermore, the proposed universal quantitative method for evaluating the treatment effectiveness in patients with various types of tissues damage to the trunk and extremities makes it possible to objectively determine the level of medical service provided to each patient, which is of great importance in the context of medical service reorganization in the state.

Prospects for further research are in the field of assessing the effectiveness of surgical interventions to eliminate various types of defects in integumentary tissues and the choice of a method for reconstructing a particular wound surface based on the analysis of long-term results.

## Data Availability

The data used in writing the manuscript is part of our own clinical research and is not in the public domain.

## Personal thanks

The authors are grateful for the comprehensive support of Alma Mater for its comprehensive research support.

## References

[1] Paik JM, Pyon JK (2017). Risk Factor Analysis of Freestyle Propeller Flaps. J Reconstr Microsurg.

[2] Rehman ZU, Sophie Z, Mal L (2016). Functional outcomes after successful lower extremity arterial injuries repair. J Pak Med Assoc.

[3] Meyer A, Horch RE, Schoengart E, Beier JP (2016). Results of combined vascular reconstruction by means of AV loops and free flap transfer in patients with soft tissue defects. J Plast Reconstr Aesthet Surg.

[4] Thomsen JB, Rindom MB, Rancati A, Angrigiani C (2021). Thoracodorsal artery flaps for breast reconstruction-the variants and its approach. Arch Plast Surg.

[5] Bulla A, Delgove A, De Luca L, Pelissier P, Casoli V (2020). The esthetic outcome of lower limb reconstruction. Ann Chir Plast Esthet.

[6] Qian Y, Li G, Zang H, Cao S, Liu Y, Yang K, Mu L (2018). A Systematic Review and Meta-analysis of Free-style Flaps: Risk Analysis of Complications. Plast Reconstr Surg Glob Open.

[7] MSF Protocols and Guidelines (2016). Revised. Milano.

[8] Rasband WS, Image J (1997-2016). National Institutes of Health. http://imagej.nih.gov/ij/.

[9] Liu Z, Tang X, Wang D, Wei Z, Jin W, Deng C, Qi J (2017). [Repair of composite tissue defects and functional reconstruction of upper arm with latissimus dorsi Kiss flap]. Zhongguo Xiu Fu Chong Jian Wai Ke Za Zhi.

[10] (2018). ATLS (X Edit.), ACSCT Royal College of Surgeons of England. https://goo.gl/OfBK07.

[11] Dong Q, Gu G, Wang L, Fu K (2017). [Application of modified adjustable skin stretching and secure wound-closure system in repairing of skin and soft tissue defect]. Zhongguo Xiu Fu Chong Jian Wai Ke Za Zhi.

[12] AlMugaren FM, Pak CJ, Suh HP, Hong JP (2020). Best Local Flaps for Lower Extremity Reconstruction. Plast Reconstr Surg Glob Open.

[13] Sabapaty SR (2016). Treatment of mutilating hand injurues: an international perspective. Hand Clinics.

[14] Falola RA, Lakhiani C, Green J, Patil S (2018). Assessment of Function after Free Tissue Transfer to the Lower Extremity for Chronic Wounds Using the Lower Extremity Functional Scale. J Reconstr Microsurg.

[15] Pan X, Wang G, Lui TH (2017). Transplantation Treatment of Extensive Soft-Tissue Defects in Lower Extremities with a Combination of Cross-Bridge Flap and Combined Free-Tissue Flap Covered by Vacuum Sealing Drainage: One Case Report. Open Orthop J.

[16] Dingemans SA, Kleipool SC, Mulders MAM, Winkelhagen J (2017). Normative data for the lower extremity functional scale (LEFS). Acta Orthop.

[17] Blair MJ, Jones JD, Woessner AE, Quinn KP (2020). Skin Structure-Function Relationships and the Wound Healing Response to Intrinsic Aging. Adv Wound Care (New Rochelle).

[18] Rixen D, Steinhausen E, Sauerland S, Lefering R (2016). Randomized, controlled, two-arm, interventional, multicenter study on risk-adapted damage control orthopedic surgery of femur shaft fractures in multiple-trauma patients. Trials.

[19] Maurya S, Srinath N, Bhandari PS (2017). Negative pressure wound therapy in the management of mine blast injuries of lower limbs: Lessons learnt at a tertiary care center. Med J Armed Forces India.

[20] Perkins ZB, Yet B, Glasgow S, Cole E (2015). Meta-analysis of prognostic factors for amputation following surgical repair of lower extremity vascular trauma. Br J Surg.

[21] Stekelenburg CM, Marck RE, Verhaegen PDHM, Marck KW, van Zuijlen PPM (2017). Perforator-based flaps for the treatment of burn scar contractures: a review. Burns Trauma.

[22] Philandrianos C, Moullot P, Gay AM, Bertrand B (2018). Soft Tissue Coverage in Distal Lower Extremity Open Fractures: Comparison of Free Anterolateral Thigh and Free Latissimus Dorsi Flaps. J Reconstr Microsurg.

[23] Anwarul H (2017). Tissue Engineering for Artificial Organs: Regenerative Medicine, Smart Diagnostics and Personalized Medicine. Alberta.

[24] Turksen K (2017). Wound Healing: Stem Cells Repair and Restorations, Basic and Clinical Aspects.

[25] Velazquez C, Whitaker L, Pestana IA (2020). Degloving Soft Tissue Injuries of the Extremity: Characterization, Categorization, Outcomes, and Management. Plast Reconstr Surg Glob Open.

[26] Naalla R, Chauhan S, Dave A, Singhal M (2018). Reconstruction of post-traumatic upper extremity soft tissue defects with pedicled flaps: An algorithmic approach to clinical decision making. Chin J Traumatol.

[27] Hayashida K, Akita S (2017). Surgical treatment algorithms for post-burn contractures. Burns Trauma.

[28] Ebrahimi A, Nejadsarvari N, Ebrahimi A, Rasouli HR (2017). Early Reconstructions of Complex Lower Extremity Battlefield Soft Tissue Wounds. World J Plast Surg.

[29] Liu MD, Yang XK, Han F, Fang ZQ (2018). [Strategy for wound repair of skin and soft tissue defect and systematic rehabilitation treatment for functional reconstruction of patients with severe burn or trauma on knees]. Zhonghua Shao Shang Za Zhi.

[30] Macdermid JC, Khadilkar L, Birmingham TB, Athwal GS (2015). Validity of the QuickDASH in patients with shoulder-related disorders undergoing surgery. J Orthop Sports Phys Ther.

[31] Mehta SP, Fulton A, Quach C, Thistle M, Toledo C, Evans NA (2016). Measurement Properties of the Lower Extremity Functional Scale: A Systematic Review. J Orthop Sports Phys Ther.

[32] Pfeifer R, Pape HC (2016). Diagnostik und Versorgungsstrategien beim polytraumatisierten Patienten [Diagnostics and treatment strategies for multiple trauma patients]. Chirurg.

[33] Bourguignon LY (2014). Matrix hyaluronan-activated CD44 signaling promotes keratinocyte activities and improves abnormal epidermal functions. Am J Pathol.

[34] Shatirishvili M, Burk AS, Franz CM, Pace G, Kastilan T, Breuhahn K, Hinterseer E, Dierich A, Bakiri L, Wagner EF, Ponta H, Hartmann TN, Tanaka M, Orian-Rousseau V (2016). Epidermal-specific deletion of CD44 reveals a function in keratinocytes in response to mechanical stress. Cell Death Dis.

[35] Bourguignon LY, Wong G, Xia W, Man MQ, Holleran WM, Elias PM (2013). Selective matrix (hyaluronan) interaction with CD44 and RhoGTPase signaling promotes keratinocyte functions and overcomes age-related epidermal dysfunction. J Dermatol Sci.

